# Bisphenol A Release from Fiber-Reinforced vs. Conventional Stainless-Steel Fixed Retainers: An In Vitro Study

**DOI:** 10.3390/jfb16020068

**Published:** 2025-02-17

**Authors:** Efthimia Tsoukala, Niki Maragou, Andriani-Paraskevi Antonelaki, Nikolaos Thomaidis, Iosif Sifakakis

**Affiliations:** 1 Department of Orthodontics, School of Dentistry, National and Kapodistrian University of Athens, 11527 Athens, Greece; efforts@dent.uoa.gr; 2 Laboratory of Analytical Chemistry, Department of Chemistry, National and Kapodistrian University of Athens, Panepistimioupolis Zografou, 15771 Athens, Greece; nmarag@agro.uoa.gr (N.M.); antonel@chem.uoa.gr (A.-P.A.); ntho@chem.uoa.gr (N.T.)

**Keywords:** fixed retainers, glass fiber, bisphenol A, stainless-steel retainer

## Abstract

Objectives: The objectives of this study were to investigate in vitro BPA release from two common fiberglass fixed lingual canine-to-canine retainers and to compare these amounts with those released from a conventional multistranded stainless-steel orthodontic retainer. Methods: Fifty-four recently extracted teeth were divided into groups of six teeth each, formed in an arch shape. Three different retainer types were evaluated: Ribbond, EverStick Ortho and Wildcut wire. Three identical specimens were constructed for each retainer type. BPA release was determined with validated the liquid chromatography–tandem mass spectrometry method at 1 and 24 h, as well as at 7, 14 and 30 days. The method’s limits of detection and quantification were 0.32 ng/mL and 0.96 ng/mL, respectively. A two-way mixed, repeated-measures analysis of variance with Greenhouse–Geisser correction was employed to verify the existence of any significant differences. Results: Higher levels of BPA were released from the polyethylene fiber and glass fiber retainer in comparison with the conventional retainer in the present study. The differences between the systems over time were not statistically significant at the 95% confidence level. Conclusions: In vitro BPA release during the first month did not differ between the examined retainer types. The highest BPA concentrations were observed at 1 month.

## 1. Introduction

Bisphenol A (BPA) is an organic monomer with applications in the plastic and resin industry. Its main use is the manufacturing of polycarbonate hard, clear plastic, a material found in food and beverage packaging, feeding and re-usable bottles, tableware, medical devices, pesticides, etc. It is also used in the production of epoxy resin, a material used as the inside protective lining of canned metal containers [1,2,3]. BPA contamination can be caused by a variety of materials that come in contact with food, liquid, air or soil [2] and is considered toxic for human tissues. It is absorbed through different routes (digestion, inhalation, skin contact or maternofetal transmission) in the human body and can have serious adverse effects in several organs, even at low doses [4,5]. Its ability to act like a hormone and bind to estrogen receptors leads to reproduction and fertility problems, thyroid malfuncti·on and body weight issues. It has also been related to cancer; immune, liver and cardiovascular diseases; and cognitive impairment [1,2,3,6,7,8,9].

In 2006, the European Food Safety Authority (EFSA) published the first risk assessment of BPA [1]. The Food and Drug Administration (FDA) also released its safety assessment on BPA for use in food contact applications in 2008 [6]. In 2015, the EFSA committee reduced the Tolerable Daily Intake (TDI) level from 50 micrograms per kilogram of body weight per day (µg/kg of bw/day) to a temporary TDI of 4 µg/kg of bw/day [10]. Similarly, in 2014, the FDA found that doses over 5 mg/kg bw/day are systematically toxic [6]. Since then, technical meetings and consultations for the re-evaluation of the temporary TDI of 4 µg/kg of bw/day have been repeatedly held by EFSA. The last decision of the EFSA Panel on Food Contact Materials, Enzymes and Processing Aids (CEP), based on newly available study outcomes, was to lower the previous TDI to 0.2 ng BPA/kg of body weight per day [7]. The daily expected BPA exposure for the entire population across all age groups according to the 2015 EFSA opinion far exceeds the new threshold [7]. Therefore, there are serious concerns regarding the health risk of BPA exposure for the general population, especially infants.

In the dental industry, BPA is a component of resin-based materials such as resin composite restorations and sealants [8,11,12], orthodontic adhesives [13,14], orthodontic thermoplastic retainers or aligners, and some polycarbonate brackets, although the latter are not widely used anymore [3,10,15,16,17,18,19,20]. Most resin materials do not contain pure BPA but bisphenol A-glycidyl methacrylate (Bis-GMA). BPA is either a precursor of Bis-GMA during its manufacturing phase or a by-product of these Bis-GMA resins or other oligomers, such as ethoxylated bisphenol A dimethacrylate (BisEMA), bis-dimethylaminopropyl (BisDMA), 2,2-bis-(4-(3-methacryloxypropoxy)phenyl)propane (BisPMA), and bisphenol A diglycidyl ether (BADGE) [8,11,18,21]. Leaching of BPA from dental materials may result from incomplete polymerization of the resin matrix, thermal or chemical degradation during food or beverage consumption, or from bacterial or enzymatic activity and mechanical abrasion during mastication or dental treatment procedures [8,11,22]. A recent systematic review found that the amount of substances released from dental materials is analogous to the material surface and, to a lesser extent, the material volume [23].

Both clinical and laboratory studies have shown traceable levels of BPA after bonding procedures that vary depending on the study [8,11,14,18,19]. There is a major concern about the release of bisphenol A from orthodontic retainers, fixed lingual and clear or Hawley removable appliances, as they consist of free resin surfaces and remain in the oral cavity for long periods of time [24]. Studies on BPA release from adhesives used for orthodontic retainers have shown various and sometimes contradicting results concerning BPA levels [4,25,26,27,28]. Fiber-reinforced composite (FCR) retainers are a more aesthetic alternative to conventional stainless-steel retainers. Several studies are available evaluating their strength, failure, debonding resistance and microleakage [29,30]; however, their potential BPA release has not been studied yet.

Hence, the aim of this laboratory study is to investigate the amount of BPA released from two common FCR splints (Ribbond and EverStick Ortho) and to compare these BPA levels with those released from a conventional multistranded stainless-steel fixed retainer during the first month after bonding. The null hypothesis is that the BPA release from these three different types of fixed retainers is the same.

## 2. Materials and Methods

### Bonding Procedure

Fifty-four freshly extracted human teeth were divided into 9 groups of six (2 canines or premolars and 4 lower incisors each) and embedded in an arch shape. These 9 groups were randomly divided into 3 subgroups. Each subgroup had a different canine-to-canine fixed retainer bonded: (1) polyethylene fiber (Ribbond; Ribbond Inc., Seattle, WA, USA), (2) glass fiber (EverStick ORTHO; GC, Tokyo, Japan) and (3) 0.0195-inch multistranded stainless-steel wire (Wildcut; Dentsply International Inc., York, PA, USA). All nine arches had approximately the same length, and the splinting materials were all cut to the same length of 30 mm each. All procedures were performed by the same person (E.T.) following the manufacturer’s instructions. The same amount of composite resin was used on each tooth, measured with a silicone half-sphere mini mold with a 1.5 mm radius (Mini mold large wire bonder; G&H Orthodontics Inc., Franklin, IN, USA).

The teeth in all subgroups were acid-etched with 36% orthophosphoric gel (Blue etch; PPH Cerkamed, Stalowa Wola, Poland) for 30 s, rinsed with water and air-dried, and primer/bonding agent (Transbond MIP; 3M Unitek, Monrovia, CA, USA) was applied on the acid-etched surface in a thin layer and light-cured with an LED unit (iLED; Woodpecker, Zhonghai, China). In the first subgroup, MIP primer was used to wet the Ribbond and gently massaged into the fibers, without curing. The wet Ribbond was positioned on the lingual surfaces of teeth, and two doses of composite resin designed specifically for the bonding of lingual retainers (Transbond LR; 3M Unitek, Monrovia, CA, USA) were applied on each tooth using the mini mold and light-cured for 20 s per tooth.

In the second subgroup, the fiber bundle was placed on top of the teeth (wetting with primer was not necessary). Composite resin was applied on the fiber splint in the same manner as for subgroup no. 1 and light-cured for 20 s on each tooth. For subgroup 3, the multistranded steel wire (Wildcut; Dentsply International Inc., York, PA, USA) was fitted passively on the lingual surfaces of the teeth. Composite resin application and light curing were the same as in the other subgroups. All materials used in the study are presented in Table 1.

After bonding, each specimen was rinsed with cold water for 5 s to simulate the clinical conditions after bonding (the practitioner usually uses water spray or instructs the patient to rinse their mouth after bonding procedures) [31,32]. The specimens were placed in glass vials filled with 80 mL distilled water and kept at a stable temperature of 35 °C for 1 month. A glass vial filled with the same volume of distilled water was kept under the same experimental conditions and served as the control.

Aliquots of 5 mL were taken 1 h, 24 h, 7 days, 14 days and 1 month after immersion and stored in a freezer until analysis (4 °C). The samples were analyzed by liquid chromatography coupled with triple quadrupole mass spectrometry (LC-MS/MS) to detect BPA of 30 mm each. All procedures were performed by the same person (E.T.) as indicated by the manufacturer’s instructions. The same amount of composite resin was used on each tooth, measured with a silicone half-sphere mini mold with a 1.5 mm radius, (Mini mold large wire bonder; G&H Orthodontics Inc., Franklin, IN, USA).

## 3. Chemical Analysis with LC-MS/MS Technique

### 3.1. Chemicals and Reagents

Bisphenol A standard (99.9%) was purchased from Sigma Aldrich (Wisconsin, WI, USA), and the isotope-labeled internal standard BPA-d16 (99.9%) was obtained from Supelco Bellefonte. Stock solutions of BPA and BPA-d16 (1000 μg/mL) were prepared in methanol, used for further dilutions and stored at −15 °C. Methanol (MeOH) and acetonitrile (ACN) of LC-MS grade were purchased from Merck (Darmstadt, Germany). Water used as the high-performance liquid chromatography (HPLC) solvent and for sample preparation was purified with a water system (Milli-Q; Millipore, Bedford, MA, USA). Regenerated cellulose syringe filters (RC) with 15 mm diameters and 0.2 μm pore sizes were obtained from Phenomenex (Torrance, CA, USA).

### 3.2. Sample Preparation

The samples were allowed to reach room temperature, then vortexed. Following that, an adequate amount was filtered using regenerated cellulose syringe filters with a pore size of 0.2 μm. A volume of 500 µL of the filtered sample was then spiked with 30 µL of the 200 ng/mL solution of the internal standard, and 70 µL of H_2_O was added to reach a final volume of 600 μL. The concentration of BPA-d16 in the measured solution was 10 ng/mL. The solution was vortexed and analyzed using LC-MS/MS.

### 3.3. LC–MS/MS Measurements

#### 3.3.1. LC–MS/MS Optimization

Liquid chromatography–tandem mass spectrometry (LC-MS/MS) was used to identify and quantify BPA in aquatic simulants of the in vitro study using a SCIEX QTRAP 6500+ system equipped with an IonDrive Turbo V source and electrospray ionization interface (ESI) connected to a SCIEX EXION LC AD system consisting of a binary gradient pump, autosampler, column oven and system controller. Data acquisition and processing were performed with Analyst version 1.7 and SCIEX OS version 2.1.6.59781 software, respectively.

The LC-MS/MS conditions were selected based on previously published methods for BPA determination [32] and in-house optimization experiments. Chromatographic separation was achieved using a C18 Atlantis T3 column (100 mm × 2.1 mm, 3 µm) (Waters Corporation, Milford, CT, USA) column. Preliminary liquid chromatography experiments were conducted with a mobile phase of MeOH and water using standard BPA solutions, BPA-d16, a pooled sample of simulants treated with the three different materials and the same pooled sample spiked with BPA and BPA-d16. It was observed that there were interferences close to the BPA retention time in the samples, which were resolved by using acetonitrile in the mobile phase.

#### 3.3.2. LC–MS/MS Final Conditions

The final optimum LC-MS/MS conditions included chromatographic separation with a C18 Atlantis T3 analytical column (100 mm × 2.1 mm) with a particle size 3 μm under gradient elution for a 9 min run time. The optimum applied gradient elution consisted of a mobile phase composed of acetonitrile (solvent A) and water (solvent B). The gradient started at A/B 30/70 at a 0.30 mL/min flow rate, remained for 0.5 min and linearly changed to A/B 90/10 in 6.5 min with the same flow rate. At the 7.5th min, the composition of the gradient returned to the initial A/B ratio of 30/70 at a 0.35 mL/min flow rate and remained stable until the 9th min. The column oven was set to 30 °C, and the injection volume was set to 10 μL.

The ESI was operated in negative ionization mode with the spray voltage at −4.5 kV and the temperature at 400 °C. The curtain gas that protects the ion entrance optics from ambient air and solvent droplet contamination was set at 35 psi, while the nebulizer gas (GS1) and the heater gas (GS2) were set at 40 and 60 psi, respectively. The selected reaction-monitoring (SRM) *m*/*z* ions recorded for the quantification of BPA were 227.0 > 211.8 (Q) and 227.0 > 132.8 for BPA confirmation. (C) The SRM *m*/*z* ions of BPA-d16 were 241.1 > 141.9. Table 2 presents the recorded SRMs with the optimum declustering potential (DP), collision energy (CE) and collision cell exit potential (CXP), which focuses and accelerates the product ions out of the collision cell (Q2) and into the filtering quadrupole (Q3).

### 3.4. Method Validation

The method was tested using standard solutions of BPA prepared in water, a pooled sample of simulants treated with the three different materials and the pooled sample spiked with known amounts of BPA and BPA-d16.

The linearity of the response of the LC-ESI-MS/MS system versus BPA concentration was examined with a standard calibration curve constructed by measuring standard solutions of 0.1, 1, 10 and 100 ng/mL BPA, all containing 10 ng/mL BPA-d16. A matrix-matched calibration curve was prepared with the pooled sample spiked with the target analyte at three levels: 1, 10 and 100 ng/mL. Linear regression analysis was performed using the *analyte peak area/internal standard peak area* ratio against the analyte concentration. The final linear equation of the matrix-matched calibration curve resulted after the subtraction of the signal of the unfortified sample from the signal of the fortified samples. The limit of detection (LOD) and limit of quantitation (LOQ) of the method were defined as (3.3 × SD)/b and (10 × SD)/b, respectively. SD was the standard deviation of the response of six independent replicate analyses of pooled samples fortified with BPA at a concentration of 1 ng/mL. The LOD and LOQ of the method were 0.32 and 0.96 ng/mL, respectively.

For assessment of precision and accuracy, the method was applied to the pooled sample that was spiked with BPA at three fortification levels (1, 10 and 100 ng/mL) and analyzed in multiple replicates. All spiked samples contained 10 ng/mL BPA-d16. The recovery (%R) of the method was calculated by subtracting the concentration measured in the non-spiked sample from that measured in the spiked sample, then dividing by the spiked concentration (C_ADDED_) according to Equation (1).

(1) $$\%R\;=\;\frac{C_{SPIKED\;SAMPLE}-C_{NONSPIKED\;SAMPLE}}{C_{ADDED}}\times 100$$

A two-way mixed, repeated-measures analysis of variance with Greenhouse–Geisser correction was employed to verify the existence of any significant differences between the systems over time.

## 4. Results

### 4.1. Method Performance

**Figure 1:**
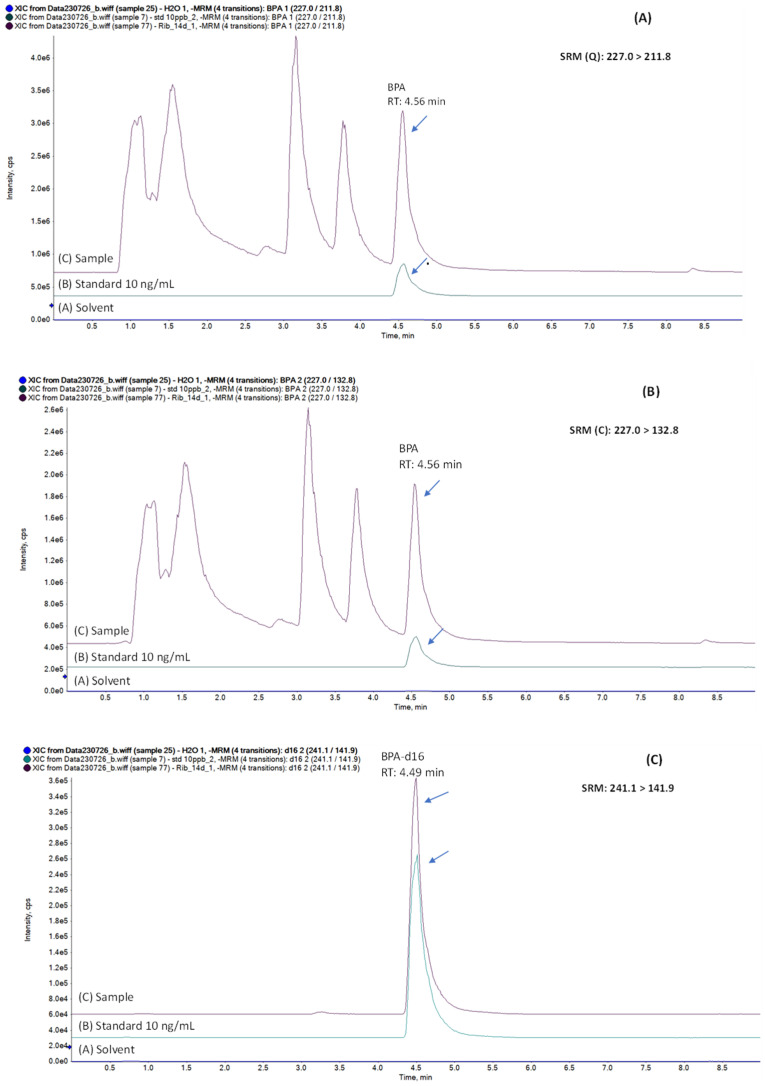
Quantification of SRM BPA (227.0 > 211.8) (**A**), confirmation of SRM BPA (227.0 > 132.8) (**B**) and SRM of BPA-d16 (**C**) for a standard solution of 10 ng/mL and the “Ribbond–14 days” sample containing 37.4 ng/mL.

Figure 1A–C illustrate the quantification and confirmation of SRM transitions (Q and C) of BPA and the SRM for BPA-d16 acquired for the solvent (H_2_O), standard solution of 10 ng/mL and the simulant of Ribbon material after 14 days incubation, which contained 37.4 ng/mL BPA.

Table 3 displays the equations of the calibration curves prepared using standards and the spiked, pooled simulant samples. The standard deviation of the slope and the intercept and the correlation coefficient of each equation are also provided. It is evident that the method exhibited linearity within the tested concentration range, with correlation coefficients exceeding 0.99. The method’s LOD and LOQ were 0.32 and 0.96 ng/mL, respectively. The recovery and precision data expressed as relative standard deviation (% RSD) are summarized in Table 4.

### 4.2. Application of the Method

**Figure 2:**
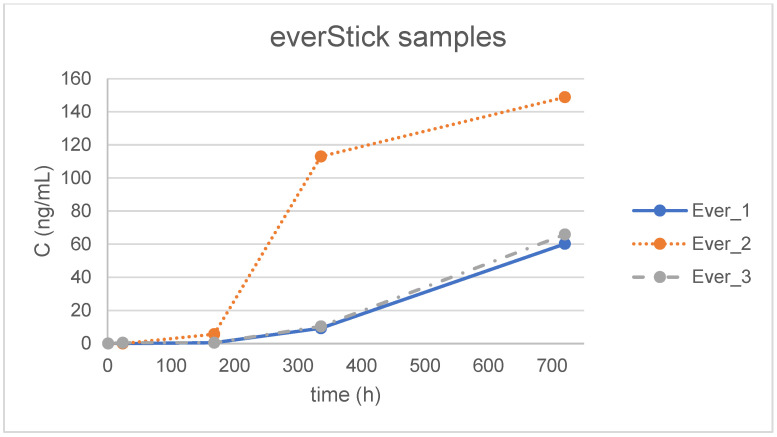
Release of BPA from EverStick samples.

**Figure 3:**
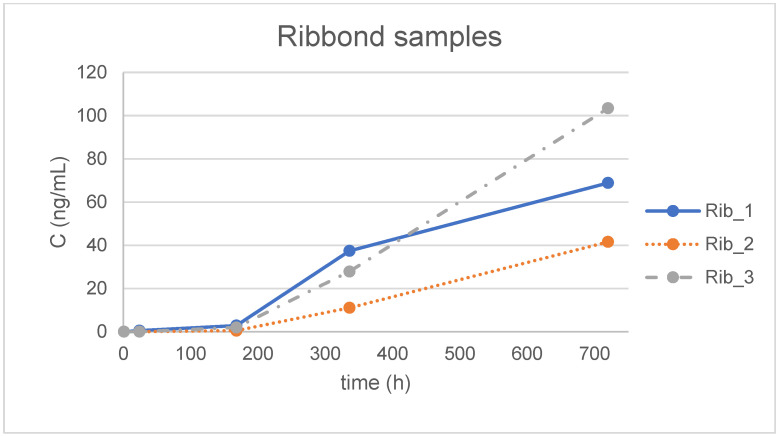
Release of BPA from Ribbond samples.

**Figure 4:**
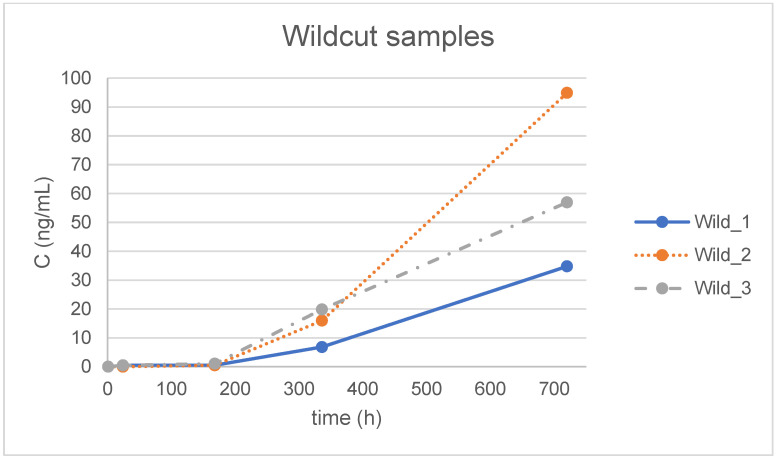
Release of BPA from Wildcut samples.

The optimized method was applied to forty-five samples of aquatic simulant incubated with different materials for various time periods (1 h, 24 h, 7 d, 14 d and 30 d) and to blank water samples treated in exactly the same way, except for the presence of the orthodontic material. BPA was not detected in the blank water samples. It is observed that the maximum determined concentration was 149 ng/mL in the simulant treated with one of the EverStick samples (Figure 2), although this concentration is above the highest matrix-matched point, which is 100 ng/mL. This result is regarded as valid, since it is of the same order of magnitude and no differentiation in linearity is anticipated between 100 and 150 ng/mL. It is noted that for the statistical analysis (Table 5 and Table 6) and the graphical presentation of the results in Figure 2, Figure 3 and Figure 4, a value of zero (0) was assigned to samples where BPA was not detected (concentration < LOD) and the numerical value of LOQ/2 was used for samples where BPA was detected but not quantified (concentration < LOQ). In more detail, Figure 2, Figure 3 and Figure 4 illustrate the concentration of BPA expressed as ng/mL versus time, expressed in hours, which was determined for the three individual replicates of the simulants treated with the three different materials, namely Ribbond, EverStick and Wildcut. Additionally, Table 5 summarizes the mean value and the corresponding standard deviation (SD) of BPA concentration (ng/mL) of the triplicate analysis of the simulants for each material for each tested time point, expressed in hours and days. Table 5 also summarizes the corresponding mean values and the standard deviations of the total mass of BPA (ng) extracted from the simulants treated with the different materials.

The mean concentration (ng/mL) and total mass (ng) of BPA release were calculated for each group (Table 5). The differences between the systems over time were not statistically significant (*p* = 0.69 for the interaction of time with the system and *p* = 0.6 for the main effect of the system) (Table 6).

## 5. Discussion

The present study was conducted in compliance with the ISO 10993-12:2012 standard [33], which standardizes the procedures for sample preparation and reference material selection for medical device testing in biological systems [34,35]. The selection of materials for testing was based on clinical relevance and the literature. A common composite resin designed specifically for the bonding of lingual retainers was used to bond three different retainer types on human teeth: polyethylene and glass fiber retainers, i.e., the two “aesthetic” retainers available in the market [29,30], as well as multistranded stainless-steel (SS) retainers, which are probably the most widely used for fixed retention. Fiber splints have an extra resin matrix to their structure, and according to the manufacturer’s instructions, more liquid resin is needed for their bonding. These facts may possibly increase bisphenol A release during clinical application. This hypothesis has not been tested yet.

A major strength of the experimental research design is that the identification of BPA was based on the signal of both SRMs, as well as quantification and confirmation at the same retention time with the standard solution. The quantification of BPA detected in the samples was performed with the matrix-matched calibration curve and the use of the deuterated internal standard to avoid over- or under-estimation of BPA content due to matrix effects. The present analysis showed that the null hypothesis is valid, i.e., in vitro BPA release during the first month did not differ between fiber-reinforced and conventional multistranded stainless-steel fixed retainers.

The orthodontic community has expressed concerns regarding BPA release, especially from lingual retainers, since a larger amount of composite is used for their construction compared to bracket bonding. Moreover, this composite resin remains exposed to the oral environment, making it vulnerable to severe abrasion. The present study is the first in the orthodontic literature to assess BPA release from fiberglass fixed retainers. Recent studies on bisphenol A release from orthodontic adhesives used for bracket bonding or removable/fixed retainers have yielded conflicting results [36,37].

Studies investigating BPA release from adhesive composites used in conventional orthodontic retainers are rather scarce and present differences in research methodology. Eliades et al. studied in vitro the amount of BPA release from Transbond XT used to bond fixed lingual retainers. The specimens were fully immersed in distilled water and analyzed with gas chromatography–mass spectroscopy. The maximum quantity of traceable BPA 1 month post immersion was 2.9 μg/L, which is far lower than in the present study [4]. This can be attributed to the higher temperature of the immersion solution used in the present study. Peloudre et al. [24] investigated monomer release from two orthodontic adhesives used for orthodontic retention: the one used in the present study (Transbond LR) and a common bracket adhesive. Gas chromatography–mass spectrometry was performed with a detection threshold of 0.02 ppm (≈0.2 mg/L). The specimens remained in distilled water for 24 h. BPA was not detected above this threshold in either of the two materials. This study investigated monomer release only in the short term, and the results showed no BPA release above the 0.02 ppm detection limit; however, a more intense release of TEGDMA was calculated with Transbond LR [26]. A further in vivo study assessed BPA release from resin composites used for orthodontic retention. Significantly higher BPA levels were detected in saliva immediately after bonding; however, these were far lower than the reference daily intake dose. All BPA levels returned to baseline after 30 days of the bonding procedure [27]. A recent systematic review concluded that the amounts of BPA released in vitro from resins used for orthodontic bonding procedures range from traces to 65.67 ppm [15].

Although several in vitro studies have investigated various adhesive composites used for orthodontic bonding procedures, there is still no consensus on their BPA release. Bationo et al. [14] studied four common orthodontic adhesives and the monomers they release. The specimens remained in distilled water for 24 h. BPA was not detected; however, the release of other monomers and rest compounds was confirmed. Another study evaluated a common bracket adhesive using different immersion media, light-curing times and methods of chemical analysis. The study lasted at least a week. BPA release was confirmed in all samples with the GC/MS method; however, when LC/MS was used, bisphenol A was only detected in ethanolsamples [13]. In a 14-day experiment, a common bracket adhesive was found to not release BPA after 24 h but only after 3 days, with a detected amount of 2.75 μg/g of material. However, the levels of BPA did not increase further after 7 and 14 days. In this study, the material samples were subjected to thermal shock treatment before the removal of the first aliquot, and the specimens were soaked in artificial saliva, not distilled water [20].

In another in vitro experiment, BPA release was detected in only one out of six evaluated common orthodontic adhesives. Its concentrations increased up to 32.10 µg/mL after the first hour, then gradually decreased to 1.7 μg/mL after 31 days. In this study, the material samples were also subjected in 20 cycles of thermal shock treatment at the beginning of the experiment, and specimens were kept in a mixture of experimental-grade water with 0.1 mL of AA poly-antibiotic mixture [38]. Conflicting results were reported from a further in vitro study that evaluated BPA release from five different orthodontic adhesives, as maximum levels were detected after 1 month. In this study, the immersion medium was an ethanol/water solution (75:25 *v*/*v*), and no thermal shocking was performed [25]. These results are consistent with the present study. A more recent study evaluated in vitro BPA release from orthodontic adhesives and the effect of brushing or mouth rinsing on BPA levels. The retainers were bonded with a common light-cured bracket adhesive. The immersion medium was distilled water, and the immersion time was 1 h. HPLC was used as the detection method. The mean BPA levels were found to be 0.2674–0.2705 µg/L, with the highest levels observed in the samples soaked in a mouth-washing solution after polymerization [39].

Two in vivo studies investigating BPA release after orthodontic bracket bonding [40,41] reported a higher level of BPA release immediately after bonding that decreased to insignificant levels the following days. A recent systematic review concluded that the amounts of bisphenol A released in vivo from orthodontic bonding resins range between 0.85 and 20.88 ng/mL, with the highest levels detected in saliva immediately after or 1 h after bonding [15].

Higher levels of BPA were released from the polyethylene fiber and glass fiber retainer compared to the conventional retainer in the present study; however, the differences were not statistically significant. Based on this evidence, these three retainer types are equally safe regarding BPA release. BPA release became more intense over time, with the maximum BPA values observed on day 30. The 24 h levels in some specimens exceeded the TDI of 0.2 ng BPA/kg per day for an adult of average weight. However, this was an in vitro study, and the entire amount of BPA was collected in the immersion media. The clinical environment is completely different, but the primary concern remains focused on the extent to which a patient swallows or absorbs this monomer. The initially higher salivary BPA content may return to baseline levels after multiple rinsings immediately after and for several hours after retainer bonding. The results of the present study clearly emphasize the importance of implementing clinical recommendations for the bonding of fixed retainers [24].

A strength of the present study is the evaluation of three specimens for each retainer type at five time points, which increased the power of the study. Additionally, we attempted to simulate the clinical conditions after bonding of a fixed retainer at the end of orthodontic treatment. Most in vitro BPA release studies in the literature evaluate only one specimen at a specific time point [14,26] or measure the release from quantities of adhesive not representative of clinical settings [13,20,25,38,39]. Further research could focus on in vivo evaluation of the BPA release of such retainers or testing of BPA-free products [42,43]. Orthodontic retainers remain in oral cavities for years or decades; however, longitudinal research is missing in this field. Moreover, the standardization of the future research methods on BPA release from dental materials is necessary for comparable results.

## 6. Conclusions

In vitro BPA release during the first month did not differ between polyethylene fiber, fiberglass and conventional multistranded stainless-steel retainers.

BPA release increased over time, with maximum BPA levels measured on day 30.

## 7. Limitations

While the present study strengthens the evidence of BPA release from fixed retainers, it also has some limitations. High standard deviations were observed within the groups, possibly due to insufficient homogenization of the distilled water, despite stirring before taking the aliquots. A mini mold was used to standardize the amount of adhesive used in each specimen; however, minor differences may have contributed to this heterogeneity. Additionally, differences in the degree of conversion of the composite could be another factor, although the light-cure tip was kept as close to the adhesive as possible in a clinical setting. Increasing the sample size or the number of observation time points may reveal more subtle differences between the tested specimens. Another limitation of this in vitro experiment is that the samples were not subjected to thermal shock cycles, which are common in the oral cavity and can affect the level of BPA released from the studied samples. Using artificial saliva as an immersion medium may have been beneficial for simulating real conditions in the oral cavity. Furthermore, retainer bonding was performed by an orthodontist who was not blinded to the groupings, although efforts were made to standardize bonding procedures.

## Figures and Tables

**Table 1:** The materials used in the present study.

Product	Manufacturer	Composition (Material Safety Data Sheet)
Transbond MIP Primer	3M Unitek	Ethyl alcohol: 30–40% (C.A.S No. 64-17-5); bisphenol-A diglycidyl ether dimethacrylate: 15–25% (C.A.S. No. 1565-94-2); 2-hydroxyethyl methacrylate: 10–20% (C.A.S No. 868-77-9); 2-hydroxy-1,3-dimethacryloxypropane: 5–15% (C.A.S No. 1830-78-0); copolymer of itaconic and acrylic acid: 5–15% (C.A.S No. 25948-33-8); diurethane dimethacrylate: 1–10% (C.A.S No. 72869-86-4); water: 1–10% (C.A.S No. 7732-18-5).
Transbond LR	3M Unitek	Silane-treated quartz: 75–85% (C.A.S. No. 100402-78-6); bisphenol A diglycidyl ether dimethacrylate (Bis-GMA): 5–15% (C.A.S. No. 1565-94-2); triethylene glycol dimethacrylate (TEGDMA): <10% (C.A.S. No. 109-16-0); silane-treated silica: <2% (C.A.S. No. 68611-44-9); N,N-dimethylbenzocaine: <0.5% (C.A.S. No. 10287-53-3).
Ribbond	Ribbond Inc.	Polyethylene (C.A.S No. 9002-88-4): 100%.
EverStick Ortho	GC Corporation Inc.	E-type glass fiber material treated with epoxy–silane-type sizing: polymethyl methacrylate (C.A.S No. 9011-14-7), 2,2-Bis [4-(2hydroxy-3-methacryloyloxy- propoxy) phenyl]-propane (C.A.S No. 1565-94-2), camphorquinone (C.A.S No. 10334-26-6): 99%, 2-dimethylaminoethyl methacrylate (C.A.S No. 2867-47-2) and hydrokinone (C.A.S No.123-31-9): 1%.
Wildcut	Dentsply International	3-strand twist flex stainless-steel wire (304-VAR).

**Table 2:** Selected reaction-monitoring (SRM) transitions of BPA and BPA-d16. Declustering potential (DP), collision energy (CE) and collision cell exit potential (CXP).

Compound	Q1 Mass (Da)	Q3 Mass (Da)	DP (V)	CE (V)	CXP (V)
BPA (Q)	227.0	211.8	−65	−24	−19
BPA (C)	227.0	132.8	−65	−34	−11
BPA-d16	241.1	141.9	−90	−34	−15

**Table 3:** Calibration curves for standard solutions and pooled simulant sample. C: ng/mL.

Matrix	Calibration Curve
Standard solutions (0.1–100 ng/mL)	y = (2104.7 ± 3.5) × 10^−4^ × C + (0.049 ± 0.018) r^2^ = 0.99998
Pooled sample (1–100 ng/mL)	y = (1443.1 ± 25.8) × 10^−4^ × C + (0.321 ± 0.116) r^2^ = 0.997

**Table 4:** Recovery and repeatability data for spiked, pooled simulant samples at three different levels.

Fortification Level (ng/mL) (Number of Replicates)	Average Recovery (%)	%RSD
1 ng/mL (*n* = 7)	135	12
10 ng/mL (*n* = 6)	114	12
100 ng/mL (*n* = 3)	70	8

**Table 5:** Concentration (ng/mL) and total mass (ng) of BPA in the 3 groups (mean, SD, *n* = 3).

Concentration (ng/mL)	1 h	24 h	7 d	14 d	30 d
Ribbond	0	0.16 (0.27)	1.74 (1.18)	25.43 (13.33)	71.29 (30.99)
EverStick	0	0.16 (0.27)	2.20 (2.98)	44.20 (59.57)	91.64 (49.56)
Wildcut	0	0.32 (0.27)	0.66 (0.31)	14.21 (6.70)	62.21 (30.40)
**Total mass (ng)**					
Ribbond	0	12 (20.78)	122 (82.57)	1653 (866.96)	4277 (1859.49)
EverStick	0	12 (20.78)	154 (209.15)	2873 (3872.60)	5499 (2973.68)
Wildcut	0	24 (20.78)	46 (22.15)	924 (435.60)	3732 (1824.11)

**Table 6:** Results from two-way mixed, repeated-measures analysis of variance.

Effect	F-Statistic	*p*-Value
System	0.556	0.6
Time	22.605	<10^−3^ *
System × Time	0.477	0.69 *

* Greenhouse-Geisser’s correction applied ($\hat{\varepsilon }$ = 0.334)

## Data Availability

The raw data supporting the conclusions of this article will be made available by the authors on request.
